# Expression in Aneuploid *Drosophila* S2 Cells

**DOI:** 10.1371/journal.pbio.1000320

**Published:** 2010-02-23

**Authors:** Yu Zhang, John H. Malone, Sara K. Powell, Vipul Periwal, Eric Spana, David M. MacAlpine, Brian Oliver

**Affiliations:** 1 Laboratory of Cellular and Developmental Biology, National Institute of Diabetes and Digestive and Kidney Diseases, National Institutes of Health, Bethesda, Maryland, United States of America; 2 Department of Pharmacology and Cancer Biology, Duke University Medical Center, Durham, North Carolina, United States of America; 3 Laboratory of Biological Modeling, National Institute of Diabetes and Digestive and Kidney Diseases, National Institutes of Health, Bethesda, Maryland, United States of America; 4 Department of Biology, Duke University, Durham, North Carolina, United States of America; Adolf Butenandt Institute, Germany

## Abstract

Extensive departures from balanced gene dose in aneuploids are highly deleterious. However, we know very little about the relationship between gene copy number and expression in aneuploid cells. We determined copy number and transcript abundance (expression) genome-wide in *Drosophila* S2 cells by DNA-Seq and RNA-Seq. We found that S2 cells are aneuploid for >43 Mb of the genome, primarily in the range of one to five copies, and show a male genotype (∼ two X chromosomes and four sets of autosomes, or 2X;4A). Both X chromosomes and autosomes showed expression dosage compensation. X chromosome expression was elevated in a fixed-fold manner regardless of actual gene dose. In engineering terms, the system “anticipates” the perturbation caused by X dose, rather than responding to an error caused by the perturbation. This feed-forward regulation resulted in precise dosage compensation only when X dose was half of the autosome dose. Insufficient compensation occurred at lower X chromosome dose and excessive expression occurred at higher doses. RNAi knockdown of the Male Specific Lethal complex abolished feed-forward regulation. Both autosome and X chromosome genes show Male Specific Lethal–independent compensation that fits a first order dose-response curve. Our data indicate that expression dosage compensation dampens the effect of altered DNA copy number genome-wide. For the X chromosome, compensation includes fixed and dose-dependent components.

## Abstract 2

Analysis of the relationship between gene copy number and gene expression in aneuploid male *Drosophila* cells reveals a global compensation mechanism in addition to X chromosome-specific dosage compensation.

## Author Summary

While it is widely recognized that mutations in protein coding genes can have harmful consequences, one can also have too much or too little of a good thing. Except for the sex chromosomes, genes come in sets of two in diploid organisms. Extra or missing copies of genes or chromosomes result in an imbalance that can lead to cancers, miscarriages, and disease susceptibility. We have examined what happens to gene expression in *Drosophila* cells with the types of gross copy number changes that are typical of cancers. We have compared the response of autosomes and sex chromosomes and show that there is some compensation for copy number change in both cases. One response is universal and acts to correct copy number changes by changing transcript abundance. The other is specific to the X chromosome and acts to increase expression regardless of gene dose. Our data highlight how important gene expression balance is for cell function.

## Introduction

The somatic cells of multicellular animals are almost exclusively diploid, with haploidy restricted to post-meiotic germ cells. Having two copies of every gene has an obvious advantage. Mutations arise de novo within cells of an organism and within organisms in populations, such that deleterious mutation-free haploid genomes are extremely rare. The wild type alleles of genes tend to be dominant to the recessive loss-of-function alleles, providing a degree of redundancy allowing diploid organisms to survive even with a substantial genetic load of deleterious mutations in each haplotype.

While the dose of most individual genes is of little consequence to the organism, larger scale genomic imbalance, or aneuploidy, is detrimental [1]–[4]. Chromosomal aneuploidy occurs when whole chromosomes are lost or duplicated and segmental aneuploidy results from deletions, duplications, and unbalanced translocations. In *Drosophila*, a systematic genome-wide segmental aneuploidy study [5] demonstrated that of all genes (now known to be about 15,000 [6]), only about 50 are haploinsufficient and just one gene is triplo-lethal. However, these same experiments showed that large deletions and duplications result in reduced viability and fertility that depends on the extent of aneuploidy, and not on any particular region or gene [5]. This indicates that the detrimental effect of aneuploidy is a collective function of multiple small effects, not a function of particular genes.

Interestingly, while aneuploidy results in inviability at the organism level, aneuploid cells can out-compete diploid cells for growth in vivo or in vitro. Human cancer cells are a good example of proliferating cells characterized by aneuploidy [7]. Most tumors are nearly diploid or tetraploid with extra or lost chromosomes. Even tumors with a normal number of chromosomes contain other rearrangements that result in segmental aneuploidy. It is likely that aneuploidy results in a systems or gene interaction defect. Given that a deleterious effect of aneuploidy is likely to occur at the level of genome balance, understanding the response to aneuploidy requires the exploration of general control mechanisms that operate at the network level.

We have turned to widely used *Drosophila* S2 tissue culture cells as an aneuploid model [8],[9]. These cells are generally tetraploid [9] and studies of gene expression and X chromosome dosage compensation indicate that they are male [10]. As a natural consequence of chromosomal sex determination in *Drosophila*, females have two X chromosomes and two pairs of autosomes (2X;2A) and males have a single X chromosome (1X;2A) [11]. Therefore, male cells can be thought of as naturally occurring chromosomal aneuploids. The response to altered gene dose probably occurs at multiple levels, but transcription is an early step in the flow of information from the genome and is a likely site for control. For example, X chromosome dosage compensation clearly occurs at the transcriptional level [12] and is exquisitely precise [13].

The Male Specific Lethal (MSL) complex regulates the balanced expression of X chromosomes in wild type 1X;2A male flies. MSL is composed of at least four major proteins (Msl1, Msl2, Msl3, and Mof) and two non-coding RNAs (RoX1 and RoX2) [11]. Mof is an acetyltransferase responsible for acetylating H4K16 [11],[14],[15]. Mof is highly enriched on the male X chromosome as a component of the MSL complex. However, Mof also associates with a more limited repertoire of autosomal genes independently of MSL [16]. H4K16ac is associated with increased transcription in many systems [17]. Therefore, it is widely believed that this acetylation results in increased expression of the X chromosome [11], although an alternative hypothesis suggests that MSL sequesters Mof from the autosomes to drive down autosome expression [18]. Determining which of these mechanisms occurs is complicated by the very nature of sampling experiments when much of the transcriptome is altered. The number of X chromosome transcripts sampled from the transcriptome depends on the relative abundance of the X chromosome and autosome transcripts. The salient feature of both models is balanced X chromosome and autosome expression.

While the term dosage compensation is used to describe X chromosome expression, dosage compensation is not restricted to X chromosomes in *Drosophila*. Autosomes also show significant, but much less precise, dosage compensation at the expression level [13],[19]–[21], suggesting that there is a general dose response genome-wide. Despite the clear role of MSL in X chromosome dosage compensation, the control system rules for MSL function and the contribution of global compensation mechanisms to the specific case of the X chromosome are poorly understood.

There are three basic transcript control mechanisms that could modify the effect of gene dose: buffering, feedback, and feed-forward [22]. Here we define buffering as the passive absorption of gene dose perturbations by inherent system properties. For example, if transcription obeys mass-action kinetics and the gene/transcription complex is considered an enzyme [23], then one would not expect a one-to-one relationship between mRNA and gene copy because of the small effect of a change in enzyme concentration at steady-state [24]. In addition to the enzymatic properties of transcription, more than a generation of molecular biologists has elegantly described extensive transcriptional regulation networks controlling key phenotypes [25]. These regulatory motifs are sensitive to changes in gene dose [26]. Feedback is an outstanding error-controlled regulator that detects deviations from the norm and implements corrective action. Feed-forward regulation differs in that it anticipates the possible effect of perturbations on the system rather than correcting the perturbation after the deviation occurs. This could operate if cells detect copy number and correct transcription levels before a quantitative error in transcript abundance is evident.

In male embryos, the sex determination hierarchy detects X chromosome number and leads to association of the MSL complex with the X chromosome before zygotic transcription is activated [27], as expected for a feed-forward regulator. However, MSL is selectively bound to transcribed genes [28], which is also consistent with feedback regulation. By examining the response of X chromosome genes to dose in the presence and absence of MSL, we show that X chromosome dosage compensation results from a combination of MSL-dependent feed-forward regulation based on anticipated effects from unbalanced gene dose and a more general and dynamic response to perceived gene dose. The latter could be due to negative feedback, buffering, or both.

## Results

### Segmental Aneuploidy in S2 Cells

**Figure 1:**
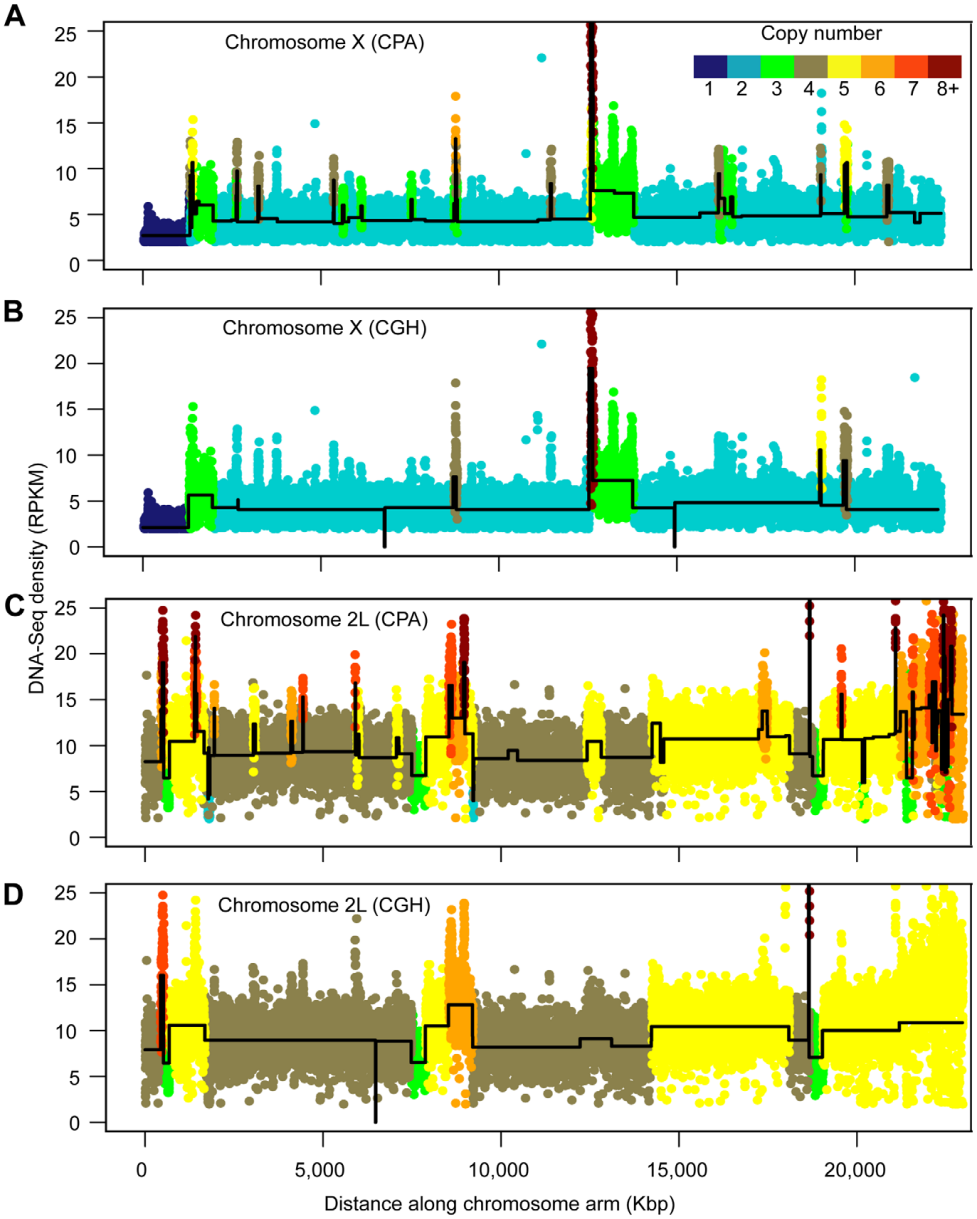
S2 cell DNA copy number. (A–D) DNA density and copy number profiles of the X chromosome (A, B) and chromosome 2L (C, D), showing copy number of aneuploidy segments along chromosome length. The RPKM DNA-Seq density in nonoverlapping 1 kb windows was plotted against the chromosome coordinates and the final deduced copy number is indicated (color key). The copy number was determined by Bayesian change point analysis (CPA) (A, C) and CGH (B, D). The CGH results are projected onto the DNA-Seq data. The average DNA densities of each aneuploid segment between predicted breakpoints (black lines) are shown.

**Figure 2:**
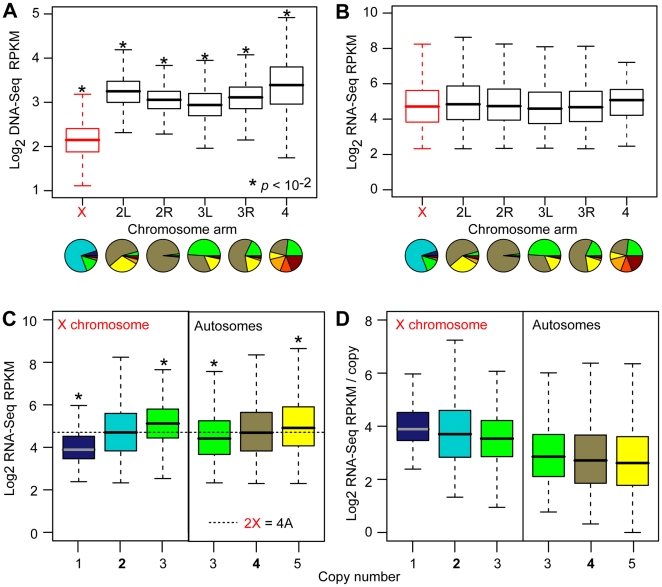
Expression at varying copy numbers. (A, B) Boxplots showing the distribution of DNA-Seq read densities (in non-overlapping 1 kb windows) mapped to chromosome arms in S2 cells (A) and the distribution of RNA-Seq expression values at the gene-level (B). Pie charts (A, B) show the distributions of copy numbers on each chromosome arm (for expressed genes only). See Figure 1 for copy number color key. The X chromosome is in red. (C, D) Boxplots showing the distribution of RNA-Seq expression values by copy number (C) and expression per copy (D). Equivalent expression medians for two copies on the X and four copies on the autosomes are indicated (dashed line). For all boxplots, the 25th to 75th percentiles (boxes), medians (lines in boxes), and ranges (whiskers, 1.5 times the interquartile range extended from both ends of the box) are shown. Asterisks indicate significant differences from all other chromosome arms (A, B) or from the 2X or 4A baseline (C).

To determine the extent of aneuploidy in S2 cells, we performed next generation sequencing (DNA-Seq) and comparative genome hybridization (CGH). These data confirmed the predicted male genotype of S2 cells, as the average sequence depth of the X chromosome (reads per kb per million reads, RPKM) was 54% of the autosome RPKM (Figures 1 and 2A).

We also found that S2 cells exhibit numerous large regions of segmental aneuploidy (Figure 1, Figure S1, Table S1). Stepwise deviations from expected dose covered ∼42% (∼40.0 Mb) of the autosomes and ∼17% (∼3.8 Mb) of the X chromosome (Figure S1). The vast majority of the aneuploid segments showed an extra or lost copy. There was high congruence between DNA-Seq and CGH methods. For example, we determined that >93% of calls for copy numbers between one and five made by DNA-Seq analysis were confirmed by CGH, even when comparing different lots of cells grown under slightly different conditions (Figure S2, Table S2). These data suggest that S2 cells are highly aneuploid but show a reasonably stable genotype. There was much more variability seen when copy number was greater than five (30% agreement between methods and cultures). This could be due to failure to call short segmental duplications or to repeat expansion/retraction in different cultures. Regardless of cause, we decided to focus our subsequent expression analyses on the high-confidence one to five copy genes (Table S3).

### Genome-Wide Compensation

We observed striking differences in DNA-Seq read density among chromosome arms due to segmental aneuploidy (Figure 2A, *p*<10^−15^, KS test). To determine if these DNA differences are also associated with similar changes at the transcript level, we profiled transcript expression by next generation sequencing (RNA-Seq). We validated RNA-Seq data by microarray profiling and found outstanding agreement (*ρ*_s_ = 0.87, *p* = 0). Expression analysis revealed striking dosage compensation. Even though copy number values significantly differed at the chromosome level (Figure 2A), we found that expression from autosome arms and the X chromosome were similar inter se (Figure 2B). In no case was the expression of a chromosome arm significantly different from all other arms (*p*>10^−2^, KS test), indicating that dosage compensation occurs genome-wide, not just on the X chromosome.

To examine the precision of dosage compensation, we determined the relationship between expression and copy number. Compensation was not perfect, as expression increased with copy number (Figure 2C, *p*<10^−4^, KS test). This imperfect compensation resulted in a sublinear relationship between copy number and gene expression, such that per copy expression values decreased with increased copy number on the autosomes and especially on the X chromosome (Figure 2D). This inverse relationship between copy number and expression per copy indicates that partial dosage compensation occurs genome-wide.

### The X Chromosome

X chromosome dosage compensation was of particular interest. In wild type males, X chromosome dose (1X) is 50% of autosomal dose (2A). In S2 cells this relationship occurred at 2X;4A due to tetraploidy. The precision of X chromosome dosage compensation in S2 cells was revealed by the indistinguishable expression of two copy X chromosome genes and four copy autosome genes (Figure 2C, *p* = 0.15, KS test). Thus X chromosome dosage compensation shows similar efficacy in diploid 1X;2A flies and in aneuploid 2X;4A tissue culture cells.

The aneuploid S2 cells also allowed us to examine the effect of X chromosome dosage compensation when the X chromosome dose was greater or less than 50%. Precise X chromosome dosage compensation did not occur at these other gene doses (Figure 2C, *p*<10^−9^, KS test). For example, when we compared expression from three copy genes on the X chromosome and autosomes, X chromosome gene expression per copy was higher despite identical copy number (Figure 2D). Thus, we suggest that X chromosome dosage compensation is error generating when the underlying X chromosome gene dose is equivalent to the autosomal gene dose. Similarly, we found under-compensated X chromosome expression when there was a single copy of an X chromosome segment. These data indicate that the anticipated or predicted X chromosome copy number that implements the sex and dosage compensation pathway determines X chromosome expression. The actual X chromosome dose is not a factor. This error generation following perturbation is a property of feed-forward regulation [22].

### MSL Complex

To evaluate the effect of the MSL complex on appropriate and error generating X chromosome dosage compensation in S2 cells, we performed RNA interference (RNAi) experiments to knockdown expression of two genes encoding key MSL components, *msl2* and *mof*. If MSL operates via feedback regulation, then knockdown should differentially alter expression depending on dose, whereas if MSL is a feed-forward regulator, the effect of MSL on expression should be X chromosome specific but dose independent.

**Figure 3:**
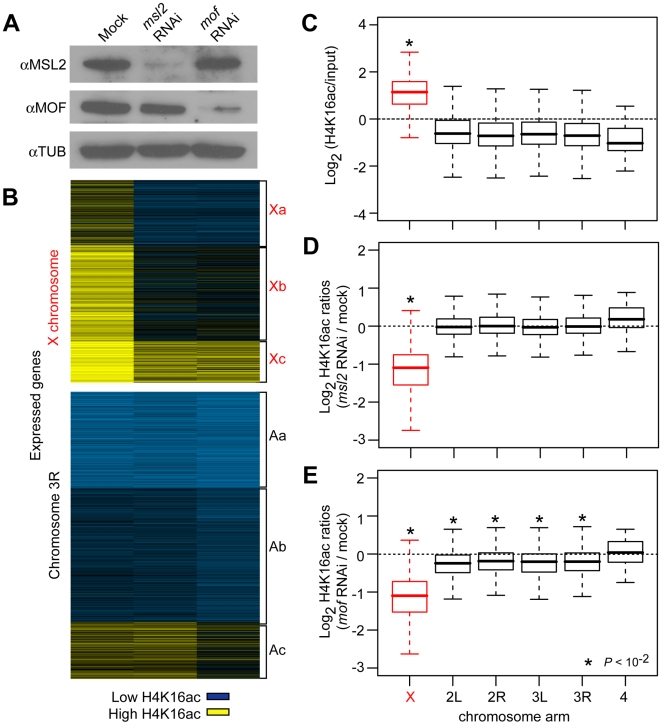
*msl2* and *mof* RNAi. (A) Western analysis showing changes in MSL protein abundance following RNAi for *msl2* and *mof* in S2 cells. (B) K-means clustering (k = 3) of H4K16ac ChIP/input ratio for expressed genes on the X chromosome and chromosome 3R in RNAi and mock treated S2 cells. Genes enriched (yellow) and depleted (blue) for H4K16ac are indicated. (C) Boxplots showing the distribution of H4K16ac ChIP/input ratios in mock treated cells for expressed genes on different chromosome arms. (D–E) Boxplots showing the distribution of H4K16ac ChIP ratios between *msl2* RNAi cells (D) or *mof* RNAi cells (E) and mock treated cells for expressed genes on different chromosome arms. Significant differences (*p*<10^−2^) among chromosome arms (C) and between RNAi and mock treated cells (D, E) are indicated by asterisks.

We selected double stranded RNAs (dsRNA) targeting *msl2* and *mof* that resulted in greater than 90% knockdown at the mRNA (not shown) and protein levels (Figure 3A). MSL is a chromatin-modifying machine. We therefore also determined if RNAi altered X chromatin. The X chromosome showed high levels of acetylation at expressed genes (Figure 3B and 3C), and both *msl2* and *mof* RNAi resulted in markedly reduced H4K16ac levels on the X chromosome as determined by chromatin immunoprecipitation on microarray (ChIP-chip, Figure 3B, 3D, and 3E). RNAi against *mof* also resulted in decreased autosomal H4K16ac (Figure 3B and 3E). All these data suggest that the RNAi treatments were effective.

**Figure 4:**
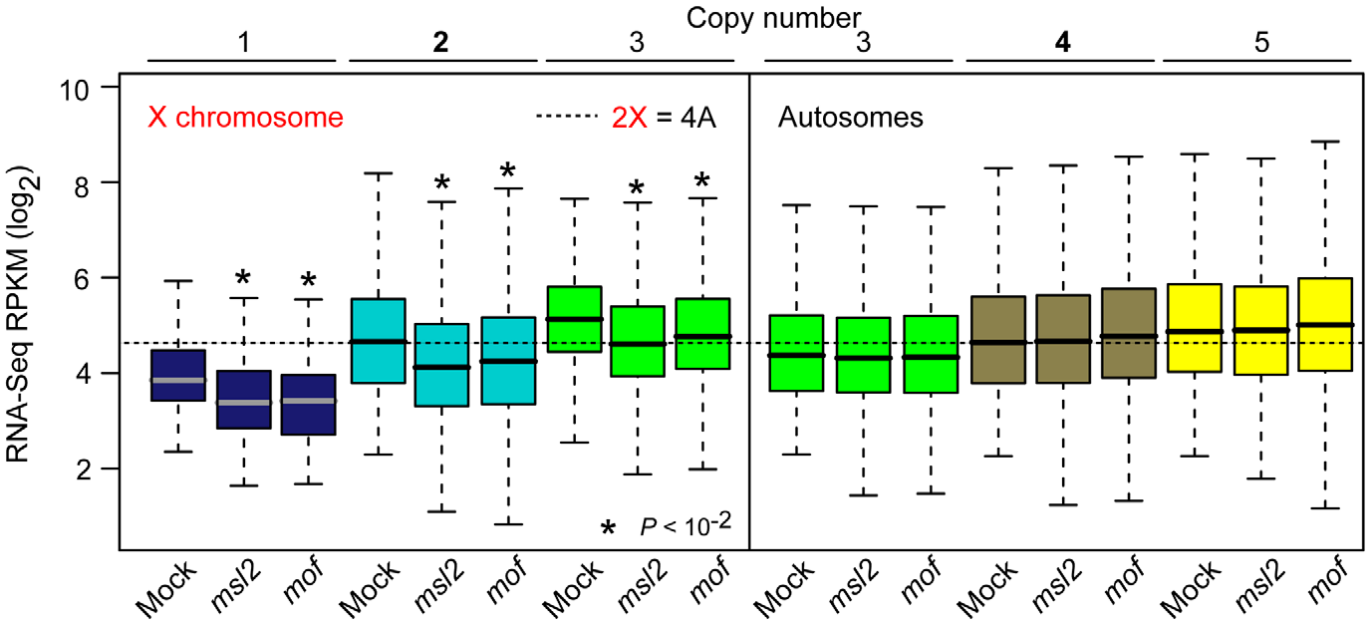
Expression following *msl2* or *mof* RNAi. Boxplots showing the distribution of expression RPKM values at indicated copy number on the X chromosome (left) and autosomes (right) in RNAi and mock treated S2 cells. Equivalent expression of two copy X chromosome genes and four copy autosomal genes in mock treated cells is shown (dashed line). See Figure 2 for boxplot format. Asterisks indicate significant expression decrease in RNAi cells compared to mock treated cells.

We then measured the effect of *msl2* and *mof* RNAi on expression by RNA-Seq. As in the previous experiments, we validated expression by microarray expression profiling and found outstanding agreement (*r*s = 0.87–0.89, *p* = 0, Figure S3). We observed decreased expression of X chromosome genes following either RNAi treatment (Figure 4, *p*<10^−2^, KS test), consistent with the role of MSL in promoting expression of X chromosome genes relative to autosomes. For example, in *mof* RNAi cells we observed a median expression of 26.4 RPKM for autosomal genes present at four copies and only 18.6 RPKM for X chromosome genes present at two copies (*p*<10^−15^, KS test). The *msl2* or *mof* RNAi treatments broke the precise equilibration of 2X with 4A expression.

**Figure 5:**
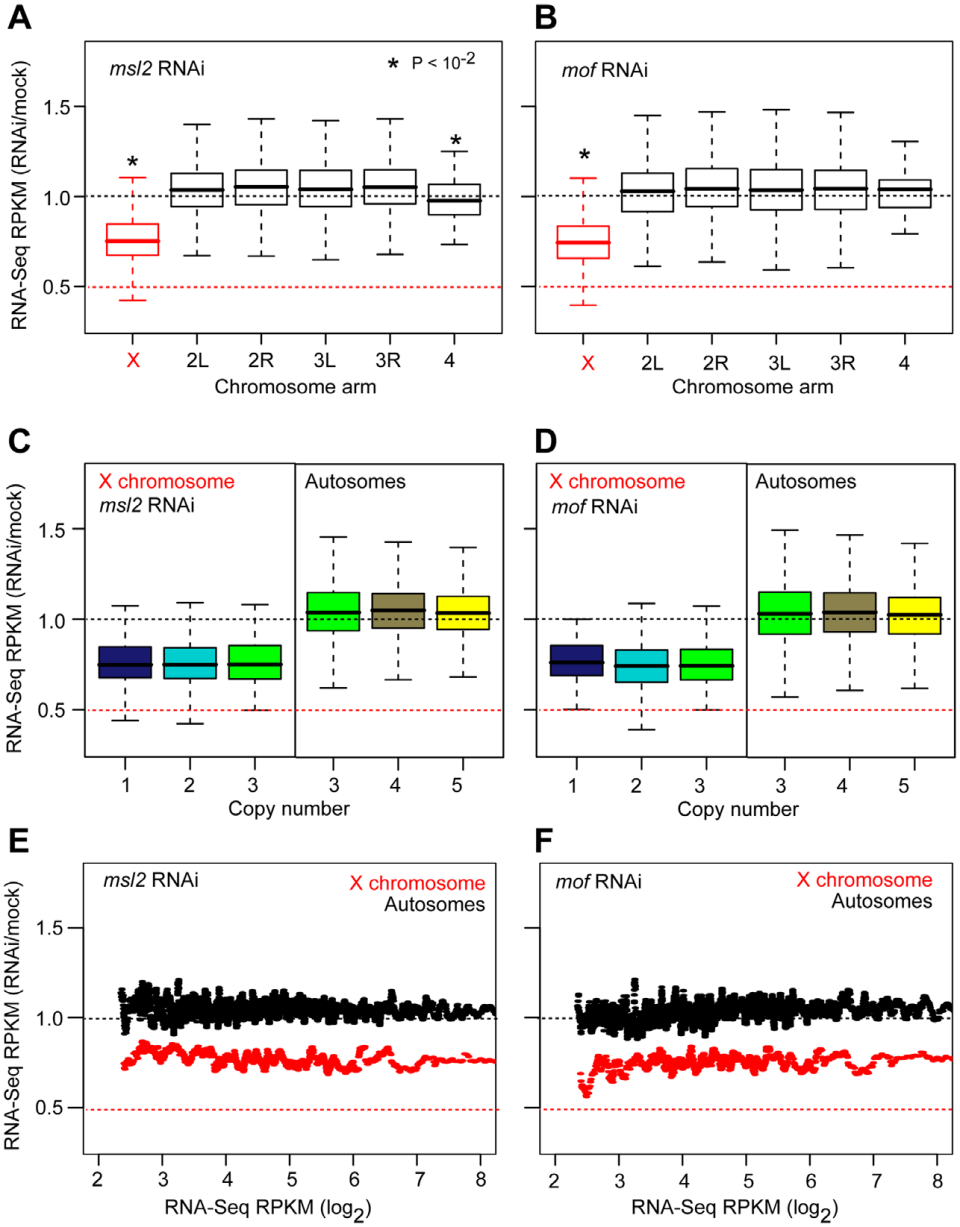
Mof and Msl2 effects on expression. (A, B) Boxplots showing the distribution of expression ratios between *msl2* RNAi cells (A) or *mof* RNAi cells (B) and mock treated cells by chromosome arms. The expected fold decrease in X chromosome expression after RNAi treatment is indicated (red dashed line). (C, D) Boxplots showing the expression ratios for *msl2* (C) and *mof* (D) RNAi treated cells at indicated gene copy numbers. The X chromosome (left) and autosomes (right) are shown separately. (E, F) The relation between gene expression and fold expression change in *msl2* (E) and *mof* (F) RNAi treated cells plotted as a moving average (20 gene/window).

We observed 1.35-fold greater X chromosome expression attributable to wild type Msl2 or Mof (average RNAi/Mock expression ratio  = 0.74, *p*<10^−15^, KS test), with little to no effect on autosomal expression (Figure 5A and 5B). If MSL acts as a strict feed-forward regulator, then MSL would have the same fold effect on all populations of X chromosome genes at a given copy number, irrespective of the actual copy number. Indeed, we observed a similar fold effect on the expression of X chromosome genes with different copy numbers (Figure 5C and 5D, 0.58<*p*<0.89 in *msl2* RNAi, 0.21<*p*<0.91 in *mof* RNAi, KS test). These data clearly indicate that MSL acts on expression based on X chromosome gene nature, rather than monitoring actual copy number.

*Drosophila* X chromosomes are dosage compensated over the full range of gene expression values. Given that MSL is bound selectively to expressed genes, we also asked if there is a relationship between expression levels and dosage compensation. We determined that the RNAi treatments had the same effect on X chromosome gene expression regardless of expression levels (Figure 5E and 5F). Interestingly, these experiments also showed only a modest effect of *mof* on autosomal expression, suggesting that the proposed autosomal function of Mof [16] is subtle. The effect of Mof on autosomes was expression level dependent, as we observed a greater fold effect at low expression levels. However, the most overt effect of wild type Msl2 or Mof was a 1.35-fold increase in X chromosome expression at all expression values. These data indicate that MSL acts as a feed-forward multiplier causing a fixed-fold effect on X chromosome expression regardless of gene copy number and basal gene expression value.

### Genome-Wide Sublinear Expression Response to Gene Dose

**Figure 6:**
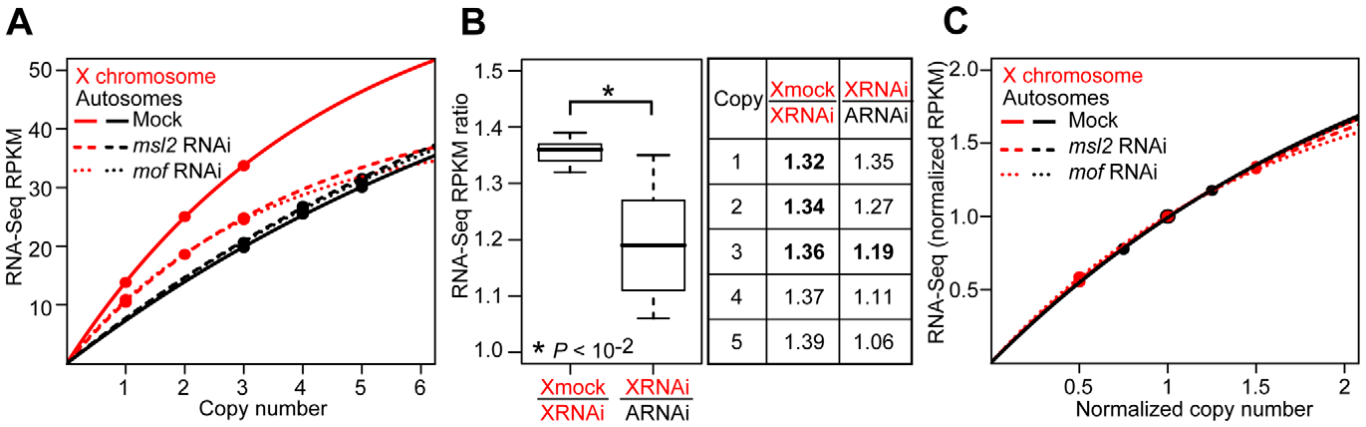
Characterization of dose-response curves. (A, C) Median expression RPKM values plotted against the DNA copy for X chromosome and autosome genes in RNAi and mock treated S2 cells based on absolute (A) or scaled (C) data. Fitted trend lines for the X chromosome (red) and autosomes (black) following mock (solid), *msl2* (dashed), and *mof* (dotted) RNAi treatment are indicated. (B) Boxplots and table showing the distribution of expression ratios among different copy numbers. Expression fold change values were calculated based on real median RPKM values (bold) or projected expression values. Asterisks indicate significant variation for the expression fold change between X chromosome and autosome genes at an equivalent dose in RNAi cells (*p*<10^−2^).

X chromosome dosage compensation is 2-fold, but we observed only a 1.35-fold effect of MSL. If MSL is the only contributor to X chromosome dosage compensation and if knockdown was complete, we would expect X chromosome and autosome genes with the same copy number to show the same expression levels following *msl2* or *mof* RNAi treatment. However, following either *msl2* or *mof* RNAi, three copy genes on the X chromosome were still 1.19-fold over-expressed relative to three copy genes on autosomes (Figure 6A, *p*<0.01, KS test). This difference between expected and observed expression could be due to residual MSL activity exclusively, or due to a combination of residual MSL activity and an MSL-independent component of X chromosome dosage compensation. The MSL-independent compensation could be the same as observed on the autosomes. Given that the fixed-fold properties of MSL also apply to residual activity, then the over-expression of X chromosome genes following RNAi treatment should also have a fixed fold effect if there is residual MSL activity. We observed significantly increased variance in the expression ratios between the X chromosome and autosomes following RNAi (*p*<10^−2^, F test, Figure 6B). This supports the idea that much of the unexplained X chromosome dosage compensation is not due to a fixed-fold effect on expression. It is possible that there are MSL-dose dependent effects on X chromosome expression due to variable affinity, although the fixed-fold effect of MSL knockdown on the population of genes makes this less likely. These data suggest that there is an MSL-independent component of X chromosome dosage compensation.

To determine if the MSL-independent component is the same dosage compensation system that operates on autosomes, we characterized the sublinear expression response to gene dose for the X chromosome and autosomes with or without RNAi treatment. There were three distinct trend lines for the relationship between copy number and expression: one for the autosomes and one each for the X chromosome with and without RNAi treatment (Figure 6A). There are an infinite number of possible sublinear curves. If the nature of the dose response on the X chromosome differed from the autosomes, or the presence or absence of MSL, then scaling should not result in a common fit. However, if the three dose response curves are the result of a common dosage compensation mechanism, then they should scale to yield a single curve that fits all three of the absolute dose-response curves.

We set median expression fold change at 2X and 4A to 1.0 for both copy number and expression (Figure 6C). We found that X chromosome and autosomes show remarkably similar fold changes in expression relative to fold changes in copy number. Additionally, the relationship between X chromosome expression and copy number is MSL independent following scaling. These data suggest that like the autosomes, the X chromosome is subject to dosage compensation based on actual gene dose. The gene dose to expression response fits a one parameter model y = x(EC50 +1)/(EC50 + x), where y is transcript abundance, x is DNA copy number expressed as a ratio relative to wild type, and EC50 is the copy number required for half maximal expression (*r* ^2^>0.99). This indicates that gene expression is a saturating function of gene dose regardless of chromosome location or the presence of MSL.

## Discussion

Our data indicate that the MSL complex and general compensation mechanisms independently contribute to male X chromosome dosage compensation. The MSL complex recognizes active X chromosome genes [28]–[31]. We have shown that MSL then acts as a simple unidirectional multiplier of expression regardless of the actual gene dose and gene expression level. In contrast, buffering and feed-back are dose sensitive and absorb the expression perturbations caused by unbalanced dose. We suggest that all these mechanisms are critical for proper X chromosome dosage compensation.

Some rough accounting illustrates the composite nature of X chromosome dosage compensation. In the *Drosophila* genus, dosage compensation results in a 2.0- to 2.2-fold increase in X chromosome expression in males relative to autosomes [13],[32]. Similarly, in S2 cells we observed a 2.08-fold increase in X chromosome expression. The fixed-fold effect of MSL resulted in at least a 1.35-fold increase in X-chromosome expression. Dose-responsive compensation also acted to increase X chromosome expression and was independent of MSL function. We can estimate the contribution of dose-responsive compensation from work performed on whole flies and on S2 cells. Autosomal dosage compensation increases per copy expression by 1.4- to 1.6-fold in diploid flies with a single copy of tens of genes [13],[19]. In agreement with those reported values, we can project that a 2-fold change in scaled DNA dose in S2 cells results in about a 1.5-fold increase in scaled gene expression. Thus, at face value, the layered effect of dose-responsive compensation and feed-forward dosage compensation may explain all of the final increase in S2 cell X chromosome expression (1.50-fold×1.35-fold = 2.03-fold).

While most work on dosage compensation focuses on the X chromosome [2],[11], other organisms also show dosage compensation on autosomes [33]. For example, mammalian trisomies show only about a 1.3-fold increase in gene expression as a result of a 1.5-fold change in gene dose [34],[35]. Compensation is likely to be a universal property of biological systems that enables cells to avoid deleterious effects of genetic load and other perturbations.

## Materials and Methods

### Cell Strains and Media

*Drosophila* S2 cells [9] (a.k.a. SL2) were obtained from *Drosophila* RNAi Screening Center (DRSC, Harvard Medical School, Boston, MA) and were grown at 25°C in Schneider's *Drosophila* Medium (Invitrogen, Carlsbad, CA) supplemented with 10% Fetal Bovine serum (SAFC Biosciences, Lenexa, KS) and Penicillin-Streptomycin (Invitrogen, Carlsbad, CA). These cells were used for all experiments, except CGH, where S2-DRSC cells were obtained from the *Drosophila* Genomics Resource Center (#181, Bloomington, IN).

### Sequencing

We extracted S2 cell genomic DNA using a genomic DNA kit (Qiagen, Valencia, CA). Approximately 2 µg of purified genomic DNA was randomly fragmented to less than 1,000 bp by 30 min sonication at 4°C with cycles of 30 s pulses with 30 s intervals using the Bioruptor UCD 200 and a refrigerated circulation bath RTE-7 (Diagenode, Sparta, NJ). Sonicated chromatin (see ChIP protocol) was purified by phenol/chloroform extraction.

We extracted S2 cell total RNA with Trizol (Invitrogen, Carlsbad, CA) and isolated mRNA using Oligotex poly(A) (Qiagen, Valencia, CA). The number of cells used for each extraction was counted using a haemocytometer. The quality of mRNA was examined by RNA 6000 Nano chip on a Bioanalyzer 2100 (Agilent, Santa Clara, CA) according to the manufacture's protocol. One hundred ng of the extracted mRNA was then fragmented in fragmentation buffer (Ambion, Austin, TX) at 70°C for exactly 5 min. The first strand cDNA was then synthesized by reverse transcriptase using the cleaved mRNA fragments as template and high concentration (3 µg) random hexamer Primers (Invitrogen, Carlsbad, CA). After the first strand was synthesized, second strand cDNA synthesis was performed using 50U DNA polymerase I and 2U RNaseH (Invitrogen, Carlsbad, CA) at 16°C for 2.5 h.

Deep sequencing of both DNA and short cDNA fragments were performed [36],[37]. Libraries were prepared according to instructions for genomic DNA sample preparation kit (Illumina, San Diego, CA). The library concentration was measured on a Nanodrop spectrophotometer (NanoDrop products, Wilmington, DE), and 4 pM of adaptor-ligated DNA was hybridized to the flow cell. DNA clusters were generated using the Illumina cluster station, followed by 36 cycles of sequencing on the Illumina Genome Analyzer, in accordance with the manufacturer's protocols. Two technical replicate libraries were constructed for each DNA-Seq sample. Two libraries were prepared from two biological replicates of each RNA material (RNAi or mock treated).

### RNAi

dsRNA for RNAi treatment [38] was produced by in vitro transcription of a PCR generated DNA template from *Drosophila* genomic DNA containing the T7 promoter sequence on both ends. Target sequences were scanned to exclude any complete 19 mer homology to other genes [39]. The dsRNAs were generated using the MEGAscript T7 kit (Ambion, Austin, TX) and purified using RNAeasy kit (Qiagen, Valencia, CA). Two different primer sets were used for each target gene, and the one with better RNAi efficiency was used for downstream experiments. The selected primer sequences for generation of *msl2* dsRNA template by PCR were as follows: forward, 5′-taatacgactcactatagggTTGCTCCGACTTCAAGACCT-3′, and reverse, 5′-taatacgactcactatagggGCATCACGTAGGAGACAGCA-3′ and the selected primer sequences for generation of *mof* dsRNA template were as follows: forward, 5′-taatacgactcactatagggGACGGTCATCACAACAGGTG-3′, and reverse, 5′-taatacgactcactatagggTGCGGTCGCTGTAGTCATAG-3′.

For RNAi treatment, S2 cells were resuspended in serum free media at 2×10^6^ cells/ml. Twenty µg dsRNA was added to 1 ml of cell suspension and incubated for 45 min at room temperature. Cells with the same serum free media treatment but without added dsRNA were used as mock treated controls. After the incubation, 3 ml complete medium was added and the cells were cultured for another 4 d. Cells were collected and split into three aliquots for mRNA extraction, chromatin immunoprecipitation, and western analysis.

### ChIP

For ChIP [40], 5–10×10^6^ S2 cells were fixed with 1% formaldehyde in tissue culture media for 10 min at room temperature. Glycine was added to a final concentration of 0.125 M to stop cross-linking. After 5 min of additional incubation and two washes with ice-cold PBS, cells were collected and resuspended in cell lysis buffer (5 mM PH 8.0 PIPES buffer, 85 mM KCl, 0.5% Nonidet P40, and protease inhibitors cocktail from Roche, Basel, Switzerland) for 10 min and then resuspended in nuclei lysis buffer (50 mM PH 8.1 Tris.HCl, 10 mM EDTA, 1% SDS and protease inhibitors) for 20 min at 4°C. The nuclear extract was sheared to 200–1,000 bp by sonication on ice for 8 min (pulsed 8 times for 30 s with 30 s intervals using a Misonix Sonicator 3000; Misonix, Inc. Farmingdale, NY). The chromatin solution was then clarified by centrifugation at 14,000 rpm for 10 min at 4°C. Five ul anti-H4AcK16 (Millipore, Billerica, MA) was incubated with the chromatin for 2 h and then was bound to protein A agarose beads at 4°C overnight. The beads were washed three times with 0.1% SDS, 1% Trition, 2 mM EDTA, 20 mM PH 8.0 Tris, and 150 mM NaCl; three times with 0.1% SDS, 1% Trition, 2 mM EDTA, 20 mM PH 8.0 Tris, and 500 mM NaCl; and twice with 10 mM PH 8.1 Tris, 1 mM EDTA, 0.25 M LiCl, 1% NP40, and 1% sodium deoxycholate. The immunoprecipitated DNA was eluted from the beads in 0.1 M NaHCO3 and 1% SDS and incubated at 65°C overnight to reverse cross-linking. DNA was purified by phenol-chloroform extraction and ethanol precipitation. The precipitated DNA for Chromatin immunoprecipitation was amplified using a Ligation-mediated PCR (LM-PCR) protocol from FlyChip [41]. ChIP was performed on triplicate biological samples.

### Microarrays

Six hundred ng of amplified DNA (ChIP enriched DNA or input DNA) were labeled using 6ug Cy3- or Cy5-labeled random nonamers (Trilink Biosciences, San Diego, CA) with 50U Klenow (New England Biolabs, Ipswich, MA) and 2 mM dNTPs. The labeled DNA was purified and hybridized to FlyGEM microarrays [42]. Arrays were scanned on an Axon 4000B scanner (Molecular Devices Corporation, Sunnyvale, CA) and signal was extracted with GenePix v.5.1 image acquisition software (Molecular Devices Corporation).

Two hundred ng aliquots of the same extracted mRNA used for RNA-Seq were labeled as described [42] and were hybridized to NimbleGen custom 12 plex microarrays at 42°C using a MAUI hybridization station (BioMicro Systems, Salt Lake City, UT) according to manufacturer instructions (NimbleGen Systems, Madison, WI). Arrays were scanned on an Axon 4200AL scanner (Molecular Devices Corporation, Sunnyvale, CA) and data were captured using NimbleScan 2.1 (NimbleGen Systems, Madison, WI).

### Western Analysis

Cell lysates were prepared from cells 4 d after dsRNA or mock treatment by boiling for 5 min in NuPAGE LDS sample buffer (Invitrogen, Carlsbad, CA). Samples were run by SDS-PAGE using a 4%–12% Bis-Tris gel (Invitrogen, Carlsbad, CA) and transferred to PVDF membrane. Blots were incubated with anti-MSL antibody (1∶500), anti-MOF antibody (1∶3,000, gifts of M. Kuroda), or anti-α tubulin antibody (1∶10,000, Sigma, St. Louis, MO) and then with HRP-secondary antibodies in PBS buffer with 0.1% Tween 20. Protein signals were detected by Pierce SuperSignal West Dura extended Duration Substrate (Thermo Fisher Scientific, Rockford, IL). Images were captured using a Fuji LAS-3000 Imager and quantified using the Image Gauge software (Fuji Film, Tokyo, Japan).

### Data Processing

Both DNA-Seq and RNA-Seq sequence reads were compiled using a manufacturer-provided computational pipeline (Version 0.3) including the Firecrest and Bustard applications [36]. Sequence reads were then aligned with the *Drosophila melanogaster* assembly (BDGP Release 5, dm3) [6],[43] using Eland. Only uniquely mapped reads with less than two mismatches were used.

For DNA-Seq data, we counted the number of reads in the non-overlapped 1 kb region along each chromosome using all sequenced reads from two technical DNA-Seq libraries and calculated the read density by the number of unique mapped reads per kb per million mapped reads (RPKM) [37]. The breakpoint positions of aneuploid segments were identified using the Bayesian analysis of change point (bcp) package from R [44]. Because some reads mapped to multiple positions in the genome and thus inappropriately lower the deduced copy number in regions with low sequence complexity, we removed all the 1 kb windows with RPKM lower than 2 (RPKM value of one copy  = 2.29) prior to change point analysis. Breakpoints with posterior possibility >0.95 were used. Copy number was assigned to segments based on the fold between average segments RPKM value between breakpoints (2.29±1.15 RPKM  = 1 copy, 4.58±1.15 RPKM  = 2 copy, etc.). Genes spanning two segments were not used in gene expression analysis.

For RNA-Seq data, we counted the number of unique mapped reads within all unique exons of *Drosophila* Flybase [45] Release 5.12 annotation (Oct. 2008) and calculated the total number of reads of all unique exons per kb of total length of unique exons per million mapped reads (RPKM) for each annotated gene. The RPKM calculation was done for individual RNA-Seq libraries separately, and then RPKM values were averaged for biological replicates (*r* ^2^ = 0.98 between replicates). Non-expressed genes are not useful for ratiometric analysis and these were therefore excluded. We used RPKM values for intergenic regions to determine expression thresholds. For intergenic regions, the RPKM values were calculated for total number of reads between adjacent gene model pairs. Only 5% of intergenic regions in S2 cells have a RPKM value greater than or equal to 4. Therefore, we called genes with RPKM values no less than 4 in S2 cells as expressed with an estimated type I error rate of 5%.

All microarray data (except CGH) and statistical tests were processed and analyzed in R/Bioconductor [46]. For the ChIP-chip experiments, we used quantile normalization based on the input channel. The distributions of raw and normalized intensities were checked to make sure that normalization was appropriate (i.e., that the skew was maintained). We used the average ChIP/input ratio from biological replicates (*r* ^2^ = 0.40–0.54 between replicates). The ChIP/input ratios in RNAi and mock treated cells were used for K-means clustering analysis with 3 nodes using Euclidean similarity metric and genes on X chromosome and autosomes were clustered separately using Cluster3.0 and then visualized using Tree-View [47]. For expression profiling, we normalized using loess within each 12-plex and quantile between 12-plexes. Average probeset log2 intensities were calculated in both channels for each gene. Correlations between array intensities and RPKM values were estimated by Spearman's rank correlation coefficient. The comparisons for the distributions of DNA densities or expression values among different chromosomes and different copy numbers were performed using two sample Kolmogorov-Smirnov tests (KS tests).

Normalization is inherently problematic when a large fraction of the genome changes expression, as in the RNAi experiments. Given that 20% of the genome is encoded on the X chromosome (X) and 80% is encoded on autosomes (A), and that one samples transcripts from a total mRNA pool to generate an expression profile, and that X chromosome expression is reduced by half and autosome expression does not change, then autosomal transcripts must be over-sampled in the experiment. Conversely, if the autosome expression is doubled, then X chromosome transcripts must be under-sampled. While it is imprudent to formally state the precise contribution of X chromosome expression changes and autosomal expression changes due to MSL-mediated dosage compensation, we can determine which makes the larger contribution based on the RPKM, total mRNA, and cell count measurements. Using this information, we calculated the log-likelihood value for two hypotheses:
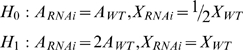



Here hypothesis *H*_0_ states that the expression of autosomes (*A*) remains the same and the expression of the X chromosome (*X*) decreases by half after RNAi treatment. Hypothesis *H*_1_ states that the expression of autosomes (*A*) is increased by 2-fold after the RNAi treatment and the expression of X chromosome (*X*) remains the same. The expected sum of expression in the RNAi treated cells is 90% of wild type for *H*_0_ and 180% for *H*_1_. E is the measured mRNA per cell. In the duplicate RNA-Seq experiments, we obtained mRNA yields of 0.16 pg and 0.17 pg/cell from mock treated, 0.15 pg and 0.19 pg/cell from *Msl2* knockdown, and 0.14 pg and 0.20 pg/cell from *Mof* knockdown S2 cells. 
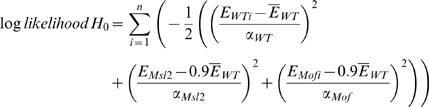


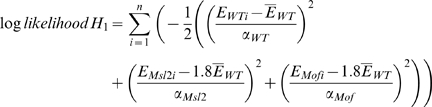



The log-likelihood of *H*_0_ – the log-likelihood of *H*_1_  = 26.4 suggests that X chromosome expression change contributes more than autosomal expression change to the observed measurements of expression in wide type cells relative to RNAi treated cells.

### Comparative Genomic Hybridization (CGH)

DNA was isolated from *Drosophila* S2-DRSC cells obtained from the *Drosophila* Genomics Resource Center (#181, Bloomington, IN) and from *w^1118^* 0–2 h embryos as described previously [48]. The isolated cell line and embryonic DNA were labeled with either Cy5 or Cy3 conjugated dUTP and subsequently hybridized to a custom Agilent genomic tiling array (GEO; GPL7787). Changes in copy number along each of the *Drosophila* chromosome arms were detected by a dynamic programming algorithm which divided each arm into the optimal number of copy number segments [49].

### Accession Numbers

All Seq and array data sets are available at GEO under accession number GSE16344. The CGH data set is available at modENCODE submission ID 596.

## Supporting Information

Figure S1 **Copy number determination by Bayesian Change Point Analysis of DNA-Seq read density.**
(1.12 MB PDF)

Figure S2 **DNA-Seq densities of each copy number defined by DNA-Seq copy number calls or CGH copy number calls.**
(0.07 MB PDF)

Figure S3 **RNA-Seq and array expression profiling.**
(2.01 MB PDF)

Table S1 **Copy number segments based on DNA-Seq.**
(0.04 MB XLS)

Table S2 **Copy number validation by DNA-Seq and CGH.**
(0.09 MB DOC)

Table S3 **The number of genes in each copy number category.**
(0.03 MB DOC)

## Abbreviations

CGH: comparative genome hybridization
ChIP: chromatin immunoprecipitation
CPA: Bayesian change point analysis
dsRNA: double stranded RNA
MSL: male specific lethal
RPKM: reads per kb per million reads
RNAi: RNA interference
