# Supplementary material for: Expression in Aneuploid Drosophila S2 Cells
Source: PLoS Biol. 2010 Feb 23;8(2):e1000320. doi: 10.1371/journal.pbio.1000320 (PMC2826376; doi:10.1371/journal.pbio.1000320)
Supplement: Figure S1 — Copy number determination by Bayesian Change Point Analysis of DNA-Seq read density. (1.12 MB PDF) [file pbio.1000320.s001.pdf]

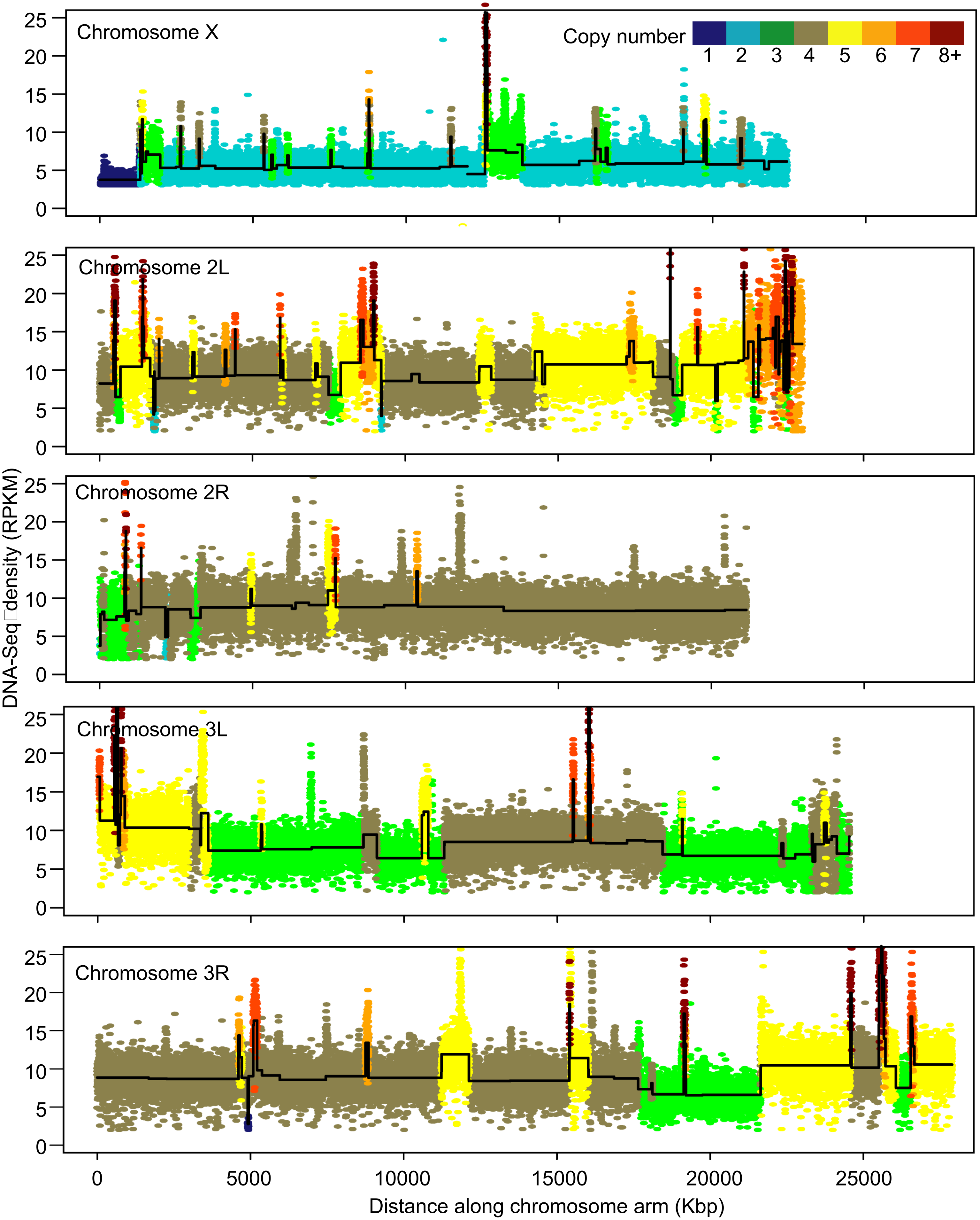

Figure S1. Copy number determination by Bayesian Change Point Analysis of DNA-seq read density. See Figure 1 for format.
