# Supplementary material for: Expression in Aneuploid Drosophila S2 Cells
Source: PLoS Biol. 2010 Feb 23;8(2):e1000320. doi: 10.1371/journal.pbio.1000320 (PMC2826376; doi:10.1371/journal.pbio.1000320)
Supplement: Figure S2 — DNA-Seq densities of each copy number defined by DNA-Seq copy number calls or CGH copy number calls. (0.07 MB PDF) [file pbio.1000320.s002.pdf]

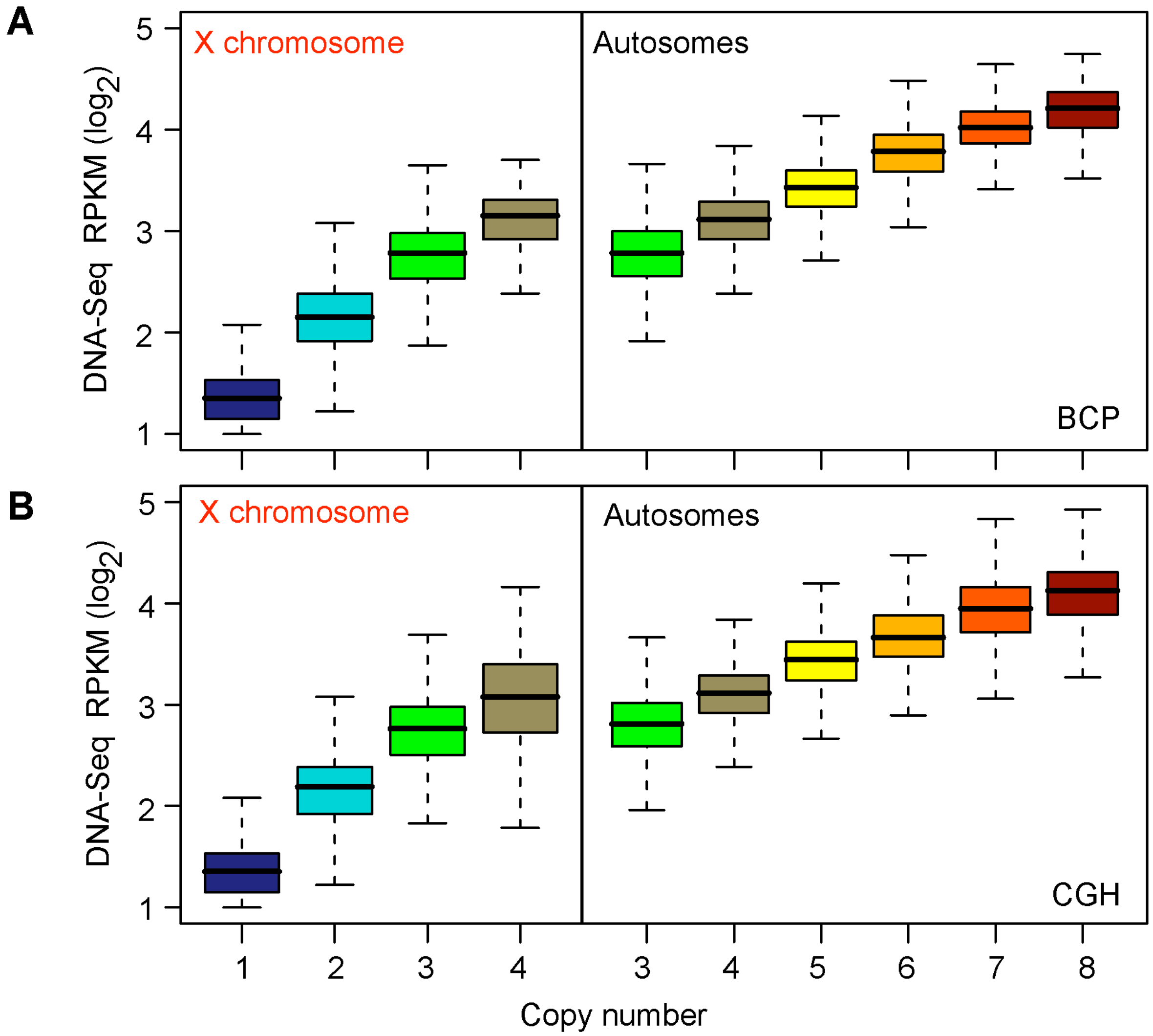

Figure S2. DNA-Seq densities of Bayesian Change Point Anaylsis (BCP) copy number calls based on DNA-Seq data (A) or Comparative Genome Hybridization (CGH) copy number calls from a separate culture (B). X chromosome genes (red) are to the left and autosomal genes to the right.
