# Supplementary material for: Expression in Aneuploid Drosophila S2 Cells
Source: PLoS Biol. 2010 Feb 23;8(2):e1000320. doi: 10.1371/journal.pbio.1000320 (PMC2826376; doi:10.1371/journal.pbio.1000320)
Supplement: Figure S3 — RNA-Seq and array expression profiling. (2.01 MB PDF) [file pbio.1000320.s003.pdf]

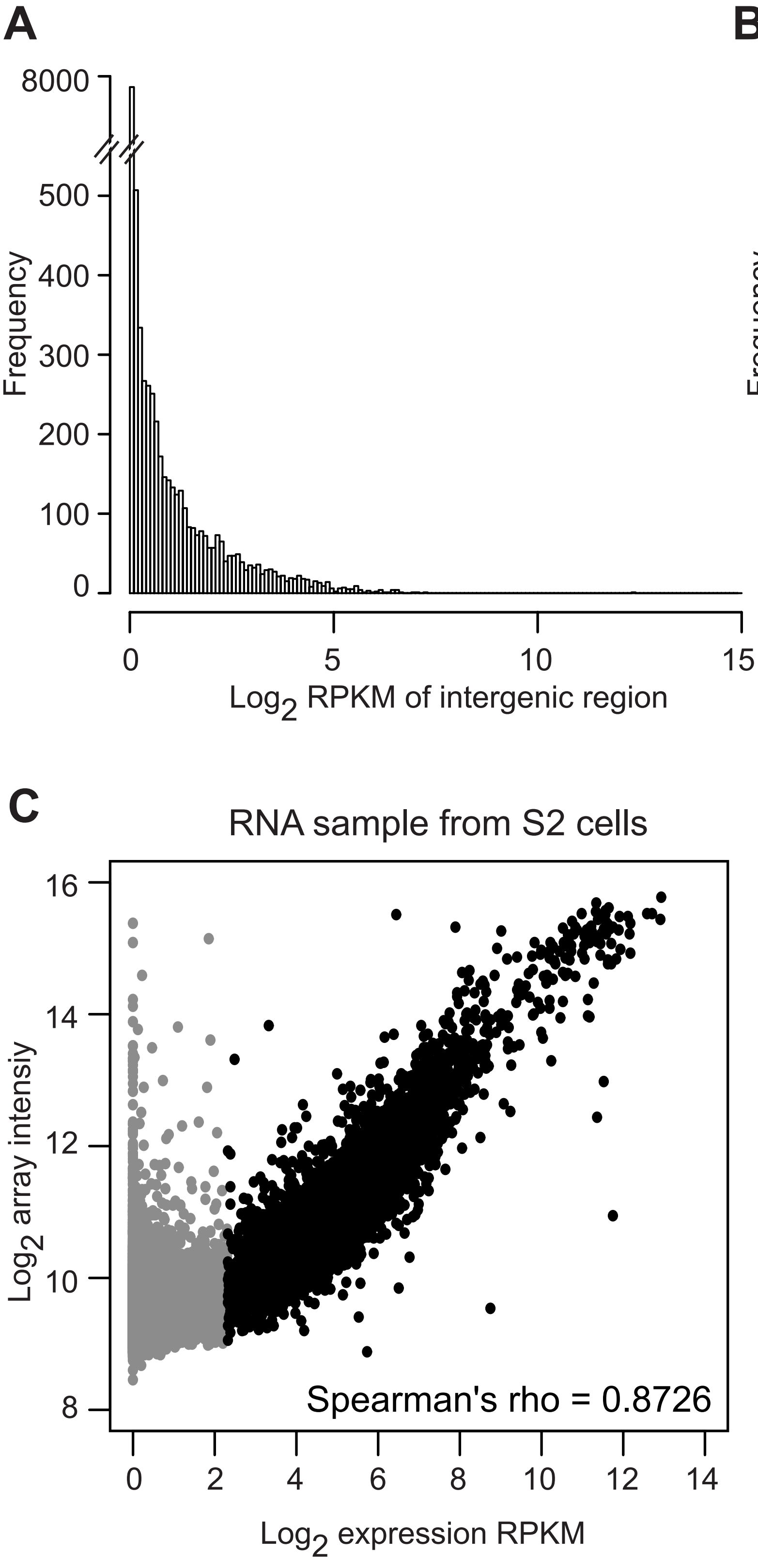

Figure S3. RNA-Seq and array expression profiling. We selected an RPKM of 4 as the threshold for expressed versus non-expressed genes based on the distribution of RPKM values in integenic regions (A) relative to transcripts (B). The agreement between expression arrays and RNA-Seq above 4 RPKM was excellent (C).
