# Supplementary material for: Expression in Aneuploid Drosophila S2 Cells
Source: PLoS Biol. 2010 Feb 23;8(2):e1000320. doi: 10.1371/journal.pbio.1000320 (PMC2826376; doi:10.1371/journal.pbio.1000320)
Supplement: Table S2 — Copy number validation by DNA-Seq and CGH. (0.09 MB DOC) [file pbio.1000320.s005.doc]

Table S2: Copy number validation by DNA-Seq and CGH.

A. Copy number validation for all expressed genes

| All chromosomes | Copy number by CGH | | | | | | | | | Total segment # | % agreement |
| --- | --- | --- | --- | --- | --- | --- | --- | --- | --- | --- | --- |
| Copy number by DNA-Seq |  | 1 copy | 2 copy | 3 copy | 4 copy | 5 copy | 6 copy | 7 copy | 8+ copy |  |  |
|  | 1 copy | 130 | 0 | 0 | 0 | 0 | 0 | 0 | 0 | 130 | 100.00 |
|  | 2 copy | 0 | 1725 | 15 | 0 | 4 | 0 | 0 | 5 | 1749 | 98.63 |
|  | 3 copy | 0 | 76 | 2218 | 49 | 112 | 10 | 11 | 11 | 2487 | 89.18 |
|  | 4 copy | 0 | 33 | 172 | 6533 | 195 | 12 | 0 | 34 | 6979 | 93.61 |
|  | 5 copy | 0 | 3 | 11 | 82 | 1631 | 35 | 4 | 33 | 1799 | 90.66 |
|  | 6 copy | 0 | 0 | 0 | 30 | 70 | 30 | 18 | 0 | 148 | 20.27 |
|  | 7 copy | 0 | 0 | 0 | 18 | 25 | 0 | 14 | 6 | 63 | 22.22 |
|  | 8+ copy | 0 | 24 | 11 | 43 | 40 | 13 | 0 | 82 | 213 | 38.50 |

B. Copy number validation for expressed genes on autosome

| Autosome | Copy number by CGH | | | | | | | | | Total segment # | % agreement |
| --- | --- | --- | --- | --- | --- | --- | --- | --- | --- | --- | --- |
| Copy number by DNA-Seq |  | 1 copy | 2 copy | 3 copy | 4 copy | 5 copy | 6 copy | 7 copy | 8+ copy |  |  |
|  | 1 copy | 0 | 0 | 0 | 0 | 0 | 0 | 0 | 0 | 0 | - |
|  | 2 copy | 0 | 0 | 0 | 0 | 0 | 0 | 0 | 0 | 0 | - |
|  | 3 copy | 0 | 23 | 2036 | 45 | 112 | 10 | 11 | 8 | 2245 | 90.69 |
|  | 4 copy | 0 | 5 | 171 | 6529 | 195 | 12 | 0 | 34 | 6946 | 94.00 |
|  | 5 copy | 0 | 3 | 10 | 79 | 1631 | 35 | 4 | 32 | 1794 | 90.91 |
|  | 6 copy | 0 | 0 | 0 | 30 | 70 | 30 | 18 | 0 | 148 | 20.27 |
|  | 7 copy | 0 | 0 | 0 | 18 | 25 | 0 | 14 | 6 | 63 | 22.22 |
|  | 8+ copy | 0 | 2 | 9 | 40 | 40 | 13 | 0 | 75 | 179 | 41.90 |

C. Copy number validation for expressed genes on X chromosome

| X chromosome | Copy number by CGH | | | | | | | | | Total segment # | % agreement |
| --- | --- | --- | --- | --- | --- | --- | --- | --- | --- | --- | --- |
| Copy number by DNA-Seq |  | 1 copy | 2 copy | 3 copy | 4 copy | 5 copy | 6 copy | 7 copy | 8+ copy |  |  |
|  | 1 copy | 130 | 0 | 0 | 0 | 0 | 0 | 0 | 0 | 130 | 100.00 |
|  | 2 copy | 0 | 1725 | 15 | 0 | 4 | 0 | 0 | 5 | 1749 | 98.63 |
|  | 3 copy | 0 | 53 | 182 | 4 | 0 | 0 | 0 | 3 | 242 | 75.21 |
|  | 4 copy | 0 | 28 | 1 | 4 | 0 | 0 | 0 | 0 | 33 | 12.12 |
|  | 5 copy | 0 | 0 | 1 | 3 | 0 | 0 | 0 | 1 | 5 | 0.00 |
|  | 6 copy | 0 | 0 | 0 | 0 | 0 | 0 | 0 | 0 | 0 | - |
|  | 7 copy | 0 | 0 | 0 | 0 | 0 | 0 | 0 | 0 | 0 | - |
|  | 8+ copy | 0 | 22 | 2 | 3 | 0 | 0 | 0 | 7 | 34 | 20.59 |

* The numbers of expressed genes with congruence copy number assignment in both DNA-seq and CGH (yellow) were indicated.
