# Supplementary material for: Expression in Aneuploid Drosophila S2 Cells
Source: PLoS Biol. 2010 Feb 23;8(2):e1000320. doi: 10.1371/journal.pbio.1000320 (PMC2826376; doi:10.1371/journal.pbio.1000320)
Supplement: Table S3 — The number of genes in each copy number category. (0.03 MB DOC) [file pbio.1000320.s006.doc]

Table S3. The number of genes in each copy number category

| **Copy number** | | **1** | **2** | **3** | **4** | **5** | **6** | **7** | **>= 8** |
| --- | --- | --- | --- | --- | --- | --- | --- | --- | --- |
| Number of genes | Autosome | 0 | 0 | 2245 | 6946 | 1794 | 148 | 64 | 179 |
|  | X chromosome | 130 | 1750 | 242 | 33 | 5 | 0 | 0 | 34 |
| Number of expressed genes (RPKM >=4) | Autosome | 0 | 0 | 1001 | 3413 | 862 | 99 | 26 | 113 |
|  | X chromosome | 58 | 892 | 170 | 29 | 1 | 0 | 0 | 26 |
